# Targeting Zoonotic Spillover Drivers for Global Pandemic Prevention: A Narrative Review

**DOI:** 10.3390/microorganisms14061316

**Published:** 2026-06-12

**Authors:** Alexandra Mpakosi, Vasileios Cholevas, Alexandra Lianou, Foteini Tziraki, Ioannis Vogiatzis, Stamatios Cholevas, Ioannis Tzouvelekis, Maria Mironidou-Tzouveleki, Konstantina A. Tsante, Deny Tsakri, Vasileios Petrakis, Petros Ioannou, Stefanos Bonovas, Rozeta Sokou, Andreas G. Tsantes

**Affiliations:** 1 Department of Immunology, General Hospital of Nikaia “Agios Panteleimon”, 18454 Piraeus, Greece; paliotziraki@hotmail.com (F.T.); jvoyia.syr@gmail.com (I.V.); 2 School of Medicine, University of Bologna, 40126 Bologna, Italy; billcholevas34@gmail.com; 3 Neonatal Intensive Care Unit, General Hospital of Nikaia “Agios Panteleimon”, 18454 Piraeus, Greece; alexlianou95@gmail.com; 4 School of Pharmacy, European University of Cyprus, Diogenes 2404, Engomi, 2404 Nicosia, Cyprus; stam17112004@gmail.com; 5 School of Agricultural Technology, Food Technology and Nutrition, Alexander Technological Educational Institute of Thessaloniki, 57400 Thessaloniki, Greece; tzouvelekisgiannis@yahoo.gr; 6 Department of Pharmacology, School of Medicine, Faculty of Health Sciences, Aristotle University of Thessaloniki, 54124 Thessaloniki, Greece; mmyronidauth@gmail.com; 7 Laboratory of Haematology and Blood Bank Unit, “Attikon” Hospital, School of Medicine, National and Kapodistrian University of Athens, 12462 Athens, Greece; ktsante@yahoo.com; 8 Department of Microbiology, Medical School, National and Kapodistrian University of Athens, 11527 Athens, Greece; denytsakris@gmail.com; 9 2nd University Department of Internal Medicine, Department of Infectious Diseases, University General Hospital Alexandroupolis, Democritus University Thrace, 691 00 Komotini, Greece; vasilispetrakis1994@gmail.com; 10 School of Medicine, University of Crete, 710 03 Heraklion, Greece; 11 Department of Biomedical Sciences, Humanitas University, 20072 Pieve Emanuele, Milan, Italy; stefanos.bonovas@hunimed.eu; 12 IRCCS Humanitas Research Hospital, 20089 Rozzano, Milan, Italy; 13 Neonatal Department, Aretaieio Hospital, National and Kapodistrian University of Athens, 11528 Athens, Greece; sokourozeta@yahoo.gr; 14 Department of Microbiology, Saint Savvas Oncology Hospital, 11522 Athens, Greece

**Keywords:** spillover event, pandemic, zoonoses, viral infection, SARS-CoV-2, influenza virus

## Abstract

Zoonoses account for the majority of recognized mammalian viral spillover events, primarily originating from bats, rodents, and primates. Human activities have significantly accelerated these transmissions. This narrative review synthesizes the evolutionary, ecological, pathogen-related, and anthropogenic drivers of viral zoonotic spillover to identify critical leverage points for pandemic prevention. A narrative literature review was conducted. The analysis focused on factors enabling animal pathogens to transform into human pathogens, examining host species, pathogen traits, human–animal interactions, and environmental impacts. Pathogen transformation depends on host traits, contact frequency, and viral characteristics. Anthropogenic drivers—including livestock expansion, the bushmeat trade, wet markets, and the exotic pet industry—significantly elevate spillover risks. Effective pandemic prevention requires targeted interventions at the wildlife–livestock–human interfaces. A holistic, multidisciplinary collaboration between national governments and international organizations is essential to mitigate future risks.

## 1. Introduction

Viruses are important pathogens with the ability to spread widely and rapidly in the human population. Viral infections sometimes cause outbreaks, epidemics, and pandemics with high rates of morbidity and mortality. Furthermore, these infections are often difficult to treat due to the ability of viruses, especially RNA viruses, to mutate frequently, which makes the development of effective vaccines and antiviral drugs a challenge [1].

Several viruses that infect humans and may cause pandemics are of zoonotic origin, as they are transmitted from other animal species through a process known as zoonotic spillover. However, the evolution of a spillover event into an epidemic or pandemic depends on many variables, including pathogen traits, host characteristics, climate change, and, to a large extent, modern human behaviors and activities. Among them, urbanization, destruction of the natural environment, globalization, global trade, poverty, food insecurity, changes in agricultural and livestock management, disruption of animal, plant and insect ecosystems, problematic surveillance systems, and illegal hunting and consumption of wildlife are of paramount importance [2].

Pandemics in the past tremendously affected the human population, caused millions of deaths and damaged the economy, society, and mental health [3]. On the other hand, recent viral epidemics such as influenza A, Zika virus, Ebola virus, and coronavirus have threatened global public health. In addition, millions of deaths due to the coronavirus disease 2019 (COVID-19) pandemic have been recorded worldwide. Furthermore, the societal disruptions caused by COVID-19 led to severe, compounding secondary crises—widely termed the “shadow pandemic”—that deeply impacted both mental health and the environment [4,5,6]. In July 2022, the World Health Organization (WHO) declared the mpox (formerly monkeypox) outbreak a Public Health Emergency of International Concern (PHEIC) [7].

Understanding the mechanism of zoonotic spillover of viruses to humans and the subsequent emergence of outbreaks, epidemics and pandemics is crucial for controlling any pandemic and preventing the emergence of a potential new one. Therefore, this narrative review aims to clarify how spillover events evolve into potential pandemics, providing critical information for diverse stakeholders, including policymakers, healthcare professionals, researchers, veterinarians, and zoo workers.

## 2. Historical and Evolutionary Drivers of Zoonotic Viral Spillover

For approximately 300,000 years, since the first appearance of humans, they have faced a multitude of viruses and viral infectious diseases. Most of the slowly evolving DNA viruses, such as *Herpesviridae*, *Papillomaviridae*, *Polyomaviridae*, and *Adenoviridae*, are probably ancient and have coevolved with their hosts [8]. For example, Herpes simplex virus type 1 (HSV-1) DNA was detected in the teeth of two children who lived during the Pleistocene, 31,600 years ago [9,10]. On the other hand, RNA viruses, with their enormous ability to mutate, but also to transmit between species, seem to have been acquired more recently during the Neolithic, when humans and domestic animals came into close contact [11]. Indeed, about 11,000 years ago, the climate became warmer and more stable compared to the previous period, creating more favorable living conditions. Additionally, during this period, humans developed permanent settlements, and there was a population increase due to extensive interbreeding between human groups and populations [12,13]. Gradually, humans evolved from hunter-gatherers to farmers and herders. This revolutionary transition caused radical changes in human ecology and immunity [14]. Daily, close proximity to animals provided the opportunity for zoonotic transmission of new pathogens such as rotavirus and influenza viruses. Therefore, many human strains have acquired many of their genes from other species, especially from domestic animals and common rodents, in the Neolithic era [15]. On the other hand, population growth and permanent settlement favored the spread of many infectious agents such as measles and rubella [13,16]. These radical changes affected the evolutionary forces acting on the human gene pool by imposing a series of heterogeneous selection pressures [13,17]. For example, the transition to agriculture caused genetic exchanges that led to the selection of protective mechanisms against specific pathogens. On the other hand, this may partly explain the high prevalence of other diseases such as autoimmune diseases today [18]. This is because the genes responsible for susceptibility to infectious diseases largely overlap those associated with autoimmune diseases. Indeed, during human history, selection has favored haplotypes carrying disease risk alleles, compensating for a possible beneficial role for another function [13,14]. Similarly, according to the “hygiene hypothesis” high exposure to pathogens in the past led to the development of defense mechanisms today, but at the same time contributed to susceptibility to autoimmune diseases as a process of balancing the immune system [19].

More zoonoses appeared in the Old World (Europe, Asia, Africa) than in the New World (The Americas), because domestic animals in the Old World harbored ancestral pathogens that were transferred to humans through close contact with them [20]. In addition, since domestic animals lived mainly in temperate regions, they had the opportunity to transmit many of their pathogens to humans. However, they also served as a conduit for the transmission of pathogens from wild animals to humans. In fact, the majority of the animal-derived viruses came from warm-blooded vertebrates (measles, mumps, rotavirus and smallpox from domestic animals, hepatitis B from apes, influenza A virus from birds) [20].

Nevertheless, temperate and tropical human zoonoses differ in the animals from which they originated. This has mainly to do with the phylogenetic proximity of the species and the ability of the pathogens to cross the species barrier [20]. For example, there is greater phylogenetic closeness between humans and Old World primates than between humans and New World primates. Thus, in addition to temperate diseases, tropical diseases also appear to have arisen from Old World primates [20].

## 3. Stages of Viral Adaptation and Zoonotic Transmission

**Figure 1:**
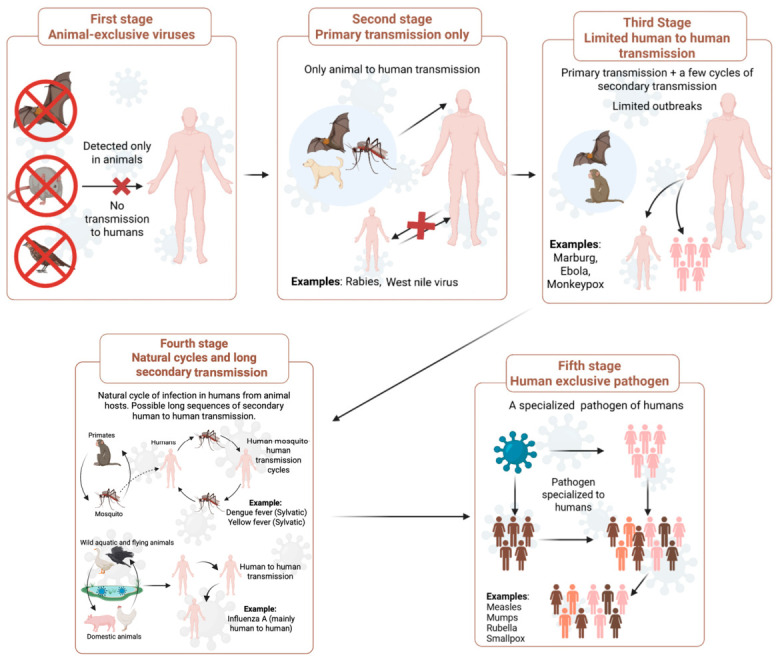
Viral evolution into human pathogens occurs through five distinct phases: Stage 1 involves pathogens restricted to animals by the species barrier. Stage 2 features primary animal-to-human spillover with no human-to-human spread. Stage 3 introduces limited secondary transmission and small outbreaks. Stage 4 establishes sustained human-to-human transmission often alongside natural sylvatic cycles. Finally, Stage 5 represents the complete adaptation of the virus into a specialized, human-exclusive pathogen. Created in BioRender. Lianou, A. (2026) https://BioRender.com/rjbdtka.

The transformation and evolution of animal pathogenic viruses into human pathogens usually follows a complex process that depends on many factors. In a first evolutionary stage, the viruses are detected exclusively in animals while there is no transmission to humans under natural conditions (inability of pathogens to cross the species barrier). This may be followed by a second stage, during which there is only animal-to-human transmission (primary transmission), but no human-to-human transmission (examples: rabies, Nipah virus, West Nile virus) [21]. In the third stage, however, in addition to the transmission of the pathogen from animals to humans, there may be secondary human-to-human transmission (a few cycles of secondary transmission between humans) and limited outbreaks (examples: Marburg, Ebola, mpox). In the fourth stage, the pathogen has a natural (sylvatic) cycle of infection of humans through primary transmission from the animal host, but there can also be long sequences of secondary transmission between humans without the involvement of animal hosts. Examples are yellow fever in which a sylvatic cycle occurs mainly rather than direct human-to-human transmission, dengue fever which is transmitted through a human–mosquito–human cycle in which infected mosquitoes acquire the virus and subsequently transmit it to susceptible human hosts and influenza A which is transmitted mainly between humans. In the fifth stage, a pathogen becomes exclusive to humans (specialized pathogen of humans). Examples of such viruses are measles, mumps, rubella, and smallpox [21] (Figure 1).

However, exclusivity in humans is difficult to fully understand. Perhaps about five million years ago, during the divergence of the chimpanzee and human lineages, an ancestral pathogen that already existed in the common ancestor of chimpanzees and humans could have co-speciated. Co-speciation could therefore be responsible for the exclusivity of certain viruses to one species of primate, but also for the appearance of related viruses in phylogenetically related primate species [11,21,22,23,24,25].

## 4. Mechanisms Driving the Transition from Spillover to Pandemic

Most viruses remain in stages 1 or 2 because they are unable to cross the species barrier (for example, rabies virus). On the other hand, as already mentioned, other animal pathogenic viruses manage to cross the species barrier and be transmitted to humans, either for only a few cycles (therefore causing only limited outbreaks before the epidemic is eliminated, as in the case of Ebola), or by evolving the ability to sustain multiple cycles of human-to-human transmission (transmission efficiency). An outbreak in an area, however, may be the result of a primary transmission followed by further secondary transmission within the human population or be due to multiple independent chains of transmission, each of which begins with a separate primary transmission from elsewhere or from a zoonotic reservoir species [26]. For example, in 2003, during the first emergence of mpox virus outside of Africa and its introduction into the United States, human cases of mpox virus infection were reported, all after human contact with infected prairie dogs. However, no human-to-human transmission was reported [27,28].

As mentioned before, phylogenetic proximity appears to play an important role since 20% of major zoonotic diseases originate from wild non-human primates (weak species barrier for pathogens to cross). For example, the phylogenetic proximity between chimpanzees and humans has resulted in a number of zoonotic diseases, including Human Immunodeficiency Virus (HIV) transmission [20,21]. On the other hand, the size of the human population, the interaction and the number of encounters between species are also important. Thus, although there is no phylogenetic proximity between rodents and humans, many diseases have been transmitted from them to humans due to their high and frequent encounters [29]. Spillover events are therefore common in locations with frequent human–animal contact, such as rural areas and wet markets (live animal markets) [30]. In the case of severe acute respiratory syndrome coronavirus 2 (SARS-CoV-2), its early detection in the densely populated city of Wuhan—where a zoonotic origin linked to live-animal markets remains a primary hypothesis—was followed by rapid urban transmission that ultimately led to the COVID-19 pandemic [31]. It is worth noting, however, that despite the fact that the weight of peer-reviewed molecular and spatial data strongly points toward a zoonotic spillover, it remains a working hypothesis rather than an indisputable fact. Since a direct progenitor virus has never been isolated in the wild, an accidental research-related incident or laboratory leak cannot be completely ruled out by existing public data [32,33,34]. Nevertheless, exposure of the host-recipient to the new pathogen can also occur indirectly through a vector or through contact, for example, with a surface containing the pathogen. This vector therefore functions as an intermediate host that mediates the transfer of the pathogen between species and can be a vertebrate host or an invertebrate vector, such as a mosquito (Table 1) [35].

### 4.1. Ecological Framework and Wildlife Reservoirs of Zoonoses

Wildlife and bushmeat are responsible for >70% of spillover events [59,60,61]. Zoonoses originating from wild animals usually follow two patterns: In the first case, once the pathogen is transmitted from animal to human, it is then easily transmitted from human to human, remaining temporarily or permanently in the human population. However, in this pattern, spillover from animals to humans occurs rarely. Examples include Marburg virus, Simian immunodeficiency virus/Human immunodeficiency virus (SIV/HIV), influenza A, Ebola, and SARS-CoV. On the other hand, according to the second pattern, the main source is a wild animal population while human-to-human transmission is rare. Such examples are rabies virus, Nipah virus and West Nile virus [62].

Most zoonoses are caused by viruses that are mainly found in primates, rodents and bats. Among them, bats are the natural reservoirs of most viral zoonoses [63]. It has been found that the number of viruses identified in bat hosts is 61 compared to 68 in rodents, despite the fact that rodent species are almost twice as numerous as bat species [64]. The high mobility of bats, due to their ability to fly, could partly contribute to the high risk of zoonotic disease transmission from them [64]. In order to adapt evolutionarily to flight, their immune system appears to have been modified by developing protective cellular mechanisms, a reduced interferon response, and a basic innate defense pathway that functionally distinguishes them from other mammals and likely allows them to carry a multitude of pathogenic microorganisms without themselves becoming ill [65,66]. Furthermore, to cope with flight, their metabolism increases excessively, producing a lot of heat, from which they are protected by having developed adaptive mechanisms that make them resistant to infections [65,67]. However, given that the spillover of pathogenic microorganisms from animals to humans depends on the opportunities for reservoir host contact, rodents should not be underestimated as important reservoirs of zoonoses, due to their population density and ecological proximity to humans [68].

Spillover events can then cause outbreaks, epidemics, and pandemics. Examples of spillover events of zoonotic viruses that successfully adapted to humans and led to pandemics are the 1918 H1N1 influenza A virus, HIV, the hepatitis C virus (HCV), and possibly SARS-CoV-2. The 1918 influenza pandemic may have been caused by spillover of the H1N1 avian influenza virus from a bird or other animal, such as pigs, the HIV was likely transmitted from chimpanzees to humans, HCV, most likely from dogs, rodents or horses (its original reservoir is however unknown), while the COVID-19 pandemic may have originated from a hypothesized spillover event of SARS-CoV-2 involving a wild reservoir and a yet-unidentified intermediate host (Table 2) [32,33,34,69].

The 1918 influenza pandemic was caused by an original influenza A H1N1 virus. The three influenza pandemics that occurred in 1957, 1968, and 2009 resulted from the reassortment of one or more genes from the original 1918 virus [76,82]. The 1977 pandemic is believed to have been caused by a strain of the H1N1 influenza virus that may have originated before 1957 and reemerged by chance [83]. The 2009 pandemic was caused by a quadruple reassortant strain of the H1N1 swine flu virus [76,77].

### 4.2. Pathogen-Specific Determinants of Adaptive Transmission

**Figure 2:**
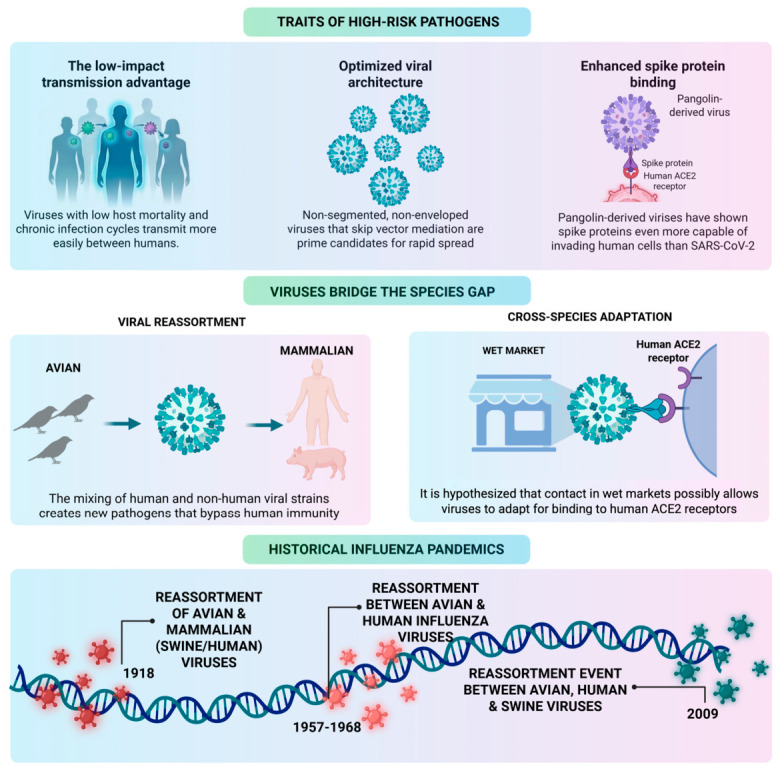
Pathogen evolution and ecological interactions serve as the primary drivers for zoonotic spillover, transforming animal-borne viruses into human pandemics. Low host mortality and non-enveloped structures enhance transmission efficiency, while mechanisms like genetic reassortment—seen in major influenza pandemics—allow viruses to bypass human immunity. Furthermore, the adaptation of SARS-CoV-2 has been proposed to reflect the potential role of wet markets and intermediate hosts in facilitating mutations of spike proteins that may enhance binding to human ACE2 receptors. Created in BioRender. L., A. (2026) https://BioRender.com/pwmdkm5.

The ability of pathogens themselves to overcome the species barrier and evolve and adapt to a new host and other tissues also plays a key role in the likelihood of a spillover event developing into an outbreak, epidemic, or pandemic. In general, viruses associated with low host mortality, those that cause long-term chronic infections, and those that are non-segmented, non-enveloped, and do not require vector mediation are likely to be transmitted more easily between humans [84]. Furthermore, the ability of viruses to develop mechanisms for long-term maintenance of secondary transmission to new hosts is of paramount importance. The influenza virus, for example, has evolved rapidly over time, causing significant epidemics with high mortality, which is mainly related to the reassortment of human and non-human influenza viruses, which results in strains to which humans have no acquired immunity [28]. For example, the 1957 and 1968 influenza pandemics resulted from reassortment between avian and human influenza viruses while the 2009 influenza pandemic from a reassortment event between avian, human and swine influenza viruses [85,86,87,88,89]. It is also likely that the 1918 pandemic influenza virus originated from a reassortment event between avian and mammalian influenza viruses, possibly swine and/or human, in the years preceding the 1918 pandemic [88,89,90]. The SARS-CoV-2 virus exhibits a high capability for human transmission. This can be partly attributed to its broad host tropism and its demonstrated transmission to various mammals, such as minks, cats, and others [91]. The coexistence of multiple susceptible species in live-animal markets, often under substandard hygiene conditions, creates potential opportunities for cross-species transmission. It is hypothesized that a cycle of transmission between bats, potential intermediate mammalian hosts—such as related carnivores—and humans may have facilitated the viral adaptation required to efficiently bind to human ACE2 receptors, potentially driving the onset of the pandemic [92,93,94]. In addition, pangolin-derived sarbecoviruses, were recently found to have spike proteins that make them even more capable than SARS-CoV-2 at invading human cells [95]. The discovery of sarbecoviruses suggests frequent and widespread spillovers from bats to other wildlife, and emphasizes the urgent need for coordinated and transnational monitoring not just of coronaviruses, but also of many other virus types [95] (Figure 2).

### 4.3. Host-Specific Susceptibility and Immunological Determinants

The vulnerability of humans is also related to the transmission of diseases. These include the host immune status, the host genetic predisposition, vaccination status, the age, and host geographic location. Individuals with severe immunosuppression and individuals who come into frequent contact with animals such as researchers, hunters, butchers, vendors, workers in forests, those who prepare meat, etc., are considered particularly vulnerable [96]. The further transmission of an infection among the human population, its severity and outcome also depend on the above factors. For example, data from the 1918 influenza pandemic, showed that despite the fact that ~500 million people worldwide were infected, the infection was not always fatal (fatality rate > 2.5%) and that a large number of people survived [97,98]. This finding, along with the paradox of the shift in case-fatality towards young adults, was likely related to the immune status of the host [99,100,101,102]. It has been hypothesized that an H1 and/or N1 influenza virus was circulating in the human population before 1889, and therefore individuals born during that period likely had cross-protective antibodies, in contrast to individuals born after 1889 who were immunologically naïve to the 1918 pandemic H1N1 virus [100,102]. However, elderly populations in remote areas, such as Indigenous people of Australia and Alaska, experienced high mortality, possibly indicating a lack of exposure to previously circulating influenza viruses that would have provided them with a protective immune response [100,102]. Interestingly, elderly people who were exposed to the 1918 influenza virus, 60–90 years before the 2009 pandemic, presented protective antibodies against infection and severe disease, which cross-reacted with the 2009 pandemic strain. Additionally, previous infections by other pathogens can also affect the host’s immune status. For example, given that the measles virus can cause a prolonged state of immunosuppression for up to 3 years after infection, young individuals who had measles infection in the years before the 1918 influenza pandemic, may have been vulnerable to severe influenza infection [102,103,104].

### 4.4. Anthropogenic Drivers and Commercial Wildlife Interfaces

Human activities such as animal husbandry practices, bushmeat trade, consumption of bushmeat, wet markets, exotic foods, ecotourism, wildlife safari, and exotic pets, have increased zoonotic virus spillover to humans [105,106,107,108,109]. For example, the international trade in wild birds caused the spread of H5N1 [110]. The bushmeat consumption has contributed to the spillover of HIV, Ebola virus, bat lyssaviruses and coronaviruses [111,112,113,114]. Especially in central Africa, zoonotic transmission of retroviruses has increased significantly due to the traditional practice of keeping primates as pets and the hunting and butchering of non-human primates. In fact, the absolute income in developing regions such as Latin America, Africa and Asia depends on bushmeat [115]. As a result, bushmeat hunting is common there. Food insecurity has actually led to ruthless exploitation of wildlife [116,117,118]. Indeed, in such areas, bushmeat meets the nutritional needs of the population. For example, loss of access has led to significant protein deficiency, stunted growth, and an increased risk of anemia in children [115]. A recent study revealed that 96% of hunters in Southeastern Zimbabwe cited their protein needs as the main motivation for illegal bushmeat hunting [119]. Given that bushmeat was sold at lower prices (2 US dollars/kg) than the average price of other meats such as beef (5 US dollars/kg), it was preferred by local communities who considered it a cheap alternative source of protein [119]. In fact, eight countries, all in sub-Saharan Africa, could be at particularly high risk of protein deficiency without wild meat consumption. However, even high-income countries such as Austria, Colombia, Denmark, Germany, New Zealand, Norway, Portugal, Switzerland, Sweden and the United States (US), have at least 1% of their protein source from wild meat. Interestingly, the US is the third largest consumer of wild meat in the world in absolute numbers [120].

Other strong motivations for illegal bushmeat hunting include the desire for increased income and unemployment [119]. Zisadza et al. showed that some hunters used illegal bushmeat hunting as their main source of income [119]. The economic benefits from illegal hunting were higher than other sources of income, such as agricultural sales, small businesses, and livestock sales [119]. In addition, in such areas, it appears that fines for illegal bushmeat hunting are very low and that the sanctions imposed are not deterrent. As a result, the majority of hunters relapse into illegal bushmeat hunting [119,121,122].

Nevertheless, as already mentioned, the bushmeat trade may be responsible for the transmission of zoonoses, both airborne and blood-borne infections during hunting and butchering, as well as diseases associated with handling and consumption [123]. Shivaprakash et al. found that among animals sold in the wildlife trade, mammals had the highest number of zoonotic viruses compared to domesticated and non-traded mammals [124]. They also found that Primates, Cetartiodactyla and Carnivores constitute the main reservoir of zoonotic viruses in the current wildlife trade, followed by Chiroptera, Rodentia, and Marsupials, which are predicted to be a significant reservoir of zoonotic viruses in the future wildlife trade [124]. This study also highlighted the role of wildlife trade in the spillover of zoonotic viruses to domestic animals, and the risk that domestic animals could potentially act as intermediate hosts for the transmission of zoonotic pathogens from traded wildlife. Examples of such zoonotic diseases transmitted to humans through domestic animals infected by wildlife species sold in markets as food or as pets, include swine flu, Middle East respiratory syndrome (MERS), and Nipah [124,125]. In 2003, monkeypox virus was introduced to the US through trade in infected rodents from Ghana. The virus was firstly transmitted to pet prairie dogs (*Cynomys ludovicianus*) that coexisted with imported African rodents and subsequently to humans [126,127,128].

A significant cluster of early COVID-19 cases was epidemiologically linked to the Huanan Seafood Wholesale Market in Wuhan. It was subsequently hypothesized that susceptible mammals sold within the market may have served as intermediate hosts or conduits, facilitating the zoonotic transmission of SARS-CoV-2 to humans from an ancestral bat coronavirus reservoir. However, because definitive physical evidence—such as an infected live animal or a direct progenitor virus—was not isolated at the site, the precise origin, the exact transmission pathway, and the specific intermediate host of SARS-CoV-2 remain subjects of ongoing scientific debate [32,33,34,129]. The severe shortage of domestic pork products in 2019, driven by the catastrophic African swine fever virus (ASFV) epizootic, caused significant supply-side shocks that may have shifted consumer demand toward alternative protein sources in Southern China. This economic shift potentially accelerated the commercial trade and consumption of farmed wildlife species, thereby increasing human and animal contact at the wildlife–livestock interface during the critical period preceding the emergence of COVID-19 [95]. Among them, civet cats, foxes, minks, and raccoon dogs were farmed for their fur and then sold at animal markets or sold live for consumption [130]. In 2002/2003, palm civets may have acted as intermediate hosts for the transmission of SARS-CoV from bats to humans. Similarly, in 2012, dromedary camels may have played the same role for MERS-CoV (Table 3) [131].

### 4.5. Environmental Encroachment and Climate-Driven Vector Dynamics

Agricultural activities can lead to the coexistence and use of the same land by multiple species such as livestock, humans and wildlife, resulting in an increased likelihood of transmission of viruses. For example, Nipah virus has been transmitted from fruit bats to pigs and horses or from fruit bats to pigs and humans. This has been attributed in part to deforestation and ecological and landscape changes that have led to an increase in pig farming and fruit tree plantations, forcing bats to invade agricultural lands [132]. On the other hand, forest encroachment, expansion of road networks, and human activities have led to the abundance of vectors and/or host density, which has contributed to the transmission of viruses such as Chikungunya virus and West Nile virus [133,134,135,136,137,138]. In addition, garbage dumps may increase the risk of disease emergence through the potential increase in population and density of animals and vectors, and the increased frequency of contact between humans, domestic animals, wildlife and vectors [139].

Urbanization has also brought water collection areas that are breeding grounds for mosquitoes, such as broken utensils, tires, flower pots, etc. For example, the global transmission of Chikungunya virus was likely due to the spread of *Aedes albopictus* through the international trade in used tires in which they had laid their eggs [52,140].

Migratory birds and ornithophilic mosquitoes may also spread mosquito-borne zoonotic avian viruses like West Nile virus to new areas [141,142]. Climate change is, however, likely to alter the distribution, composition and migration patterns of wild bird populations, resulting in subsequent changes in the transmission of viruses between wild bird species, and/or wild birds and domestic poultry [143]. In addition, climate change and global warming have also been associated with more frequent outbreaks of other viruses, such as Puumala hantavirus, through their effects on hantavirus reservoir host populations (changes in population density as well as in the species composition and geographic distribution of their reservoir hosts) [144,145]. Furthermore, high temperatures can also affect the life cycle of several vectors such as *Aedes* mosquitoes, by accelerating their maturation and reducing the incubation period of viruses, including dengue virus, within them. On the other hand, increased rainfall provides opportunities for the creation of cavities with stagnant water that mosquitoes use to lay their eggs [146,147,148].

## 5. Pandemic Prevention Frameworks and One Health Surveillance

The spillover of infectious agents from animals to humans and their transformation into human pathogens involves factors related to the evolution of the pathogens themselves, their natural habitat, and their interactions with old and new ecosystems. However, pandemics can be prevented if the competent authorities understand that it is worth spending both time and financial resources on their prevention. Therefore, it is imperative that the competent authorities remain under surveillance, anticipate changes in interfaces, and intervene to prevent the progression of the infection from a sporadic or outbreak to an epidemic and pandemic. To this end, a holistic approach is required with full and coordinated cooperation between bodies from different scientific disciplines and those involved in politics [149].

Global organizations, such as the WHO, should provide countries with guidance and protection of people from zoonoses, technical support so that they can effectively manage outbreaks and epidemics, support to improve their reporting systems and case recording, and training on clinical management, diagnosis and vector control [150].

**Figure 3:**
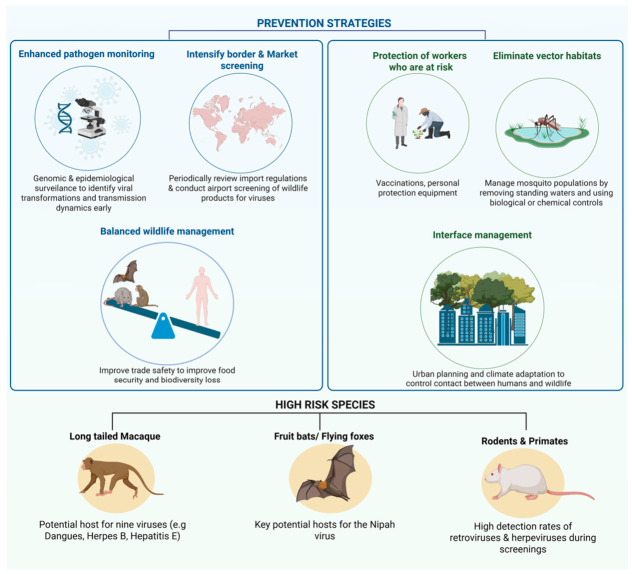
A Holistic Framework for Global Pandemic Prevention. Global health security depends on a holistic approach that integrates epidemiological surveillance with coordinated political action. By anticipating changes in wildlife–human interfaces and intervening at the sporadic outbreak stage, authorities can prevent the progression to a pandemic. Key pillars include WHO-led technical guidance, genomic monitoring of viral adaptations, and the strengthening of reporting systems to ensure early detection of zoonotic spillovers. Created in BioRender. L., A.(2026) https://BioRender.com/3t82732.

In addition, understanding the ecology and evolution of pathogens is also important for the prevention of zoonoses and the early detection of outbreaks. Epidemiological and genomic surveillance is therefore required with the aim of both the timely identification of viral factors that determine the dynamic transmission of a virus and the continuous monitoring of viral transformations and adaptation mechanisms [151] (Figure 3).

### 5.1. Mitigation Strategies for Wildlife-Associated Zoonoses

For rural communities and low-income countries whose livelihoods depend on the wildlife trade, the development of prevention strategies aimed at limiting contacts that pose the main risks of exposure to zoonoses, training strategies on monitoring, capturing, transporting wild animals, slaughtering carcasses, cooking and the consumption process, as well as preventive health strategies is required [152]. Hygiene rules must also be observed to avoid close proximity of different species of wild and domestic animals in wildlife markets, which could provide opportunities for recombination between viruses and the emergence of recombinant strains with a new phenotype [153].

Forest and zoo workers need appropriate vaccination protection against the diseases of the animals they come into contact with. In addition, there should be sufficient information from the competent authorities on how to avoid direct or indirect human contact with wild animals, providing instructions for personal protection, such as the use of protective gloves and/or a mask, careful hand washing with soap and clean water in case of handling a suspected case, and immediate seeking of medical advice from the nearest health units [154].

Surveillance methods for wildlife products need to be intensified. For example, Smith et al. reported that airport screening of rodent parts and non-human primate samples imported to the US detected retroviruses (simian foamy virus) and/or herpesviruses (cytomegalovirus and lymphocryptovirus) [155,156]. A health system should therefore periodically review import regulations, taking into account both meeting needs and ensuring a balance between the economic benefits of wildlife trade and potential risks to public health [157]. Perhaps, high-income countries that consume wild meat, could help prevent the spread of zoonoses from wildlife to humans by creating an intergovernmental body for better global surveillance and helping to direct available resources to countries where income depends on hunting and exporting wild animals [133]. International cooperation to prevent zoonoses can be further strengthened through research, timely information exchange, and the harmonization of wildlife trade regulations at the global and national levels [158].

On the other hand, removing wild meat from global food systems is not a solution, as it carries risks: The first consequence is food insecurity. In addition, to meet demand and replace wild meat, an increase in livestock farming areas is required, with consequences such as biodiversity disruption and an increased risk of infectious diseases [120,159,160]. Furthermore, since only a few groups of animals in the wildlife trade are considered reservoirs of a large number of zoonotic pathogens, attention may need to be paid only to them in order to minimize the socio-economic impact on local communities dependent on the wildlife trade [120,133,161,162,163,164]. For example, Morcatty et al. showed that the long-tailed macaque is the most commercially traded species, sold on 13/14 in Indonesian wildlife markets, and has the greatest potential to spread viral diseases, being a potential host for nine viruses, including cowpox, dengue, hepatitis E, herpes B, simian foamy, and simian retrovirus type D [165]. Short-nosed fruit bats and large flying foxes are also sold in large quantities in 10/14 markets and are potential hosts for viruses such as Nipah virus [165].

Understanding of the interfaces (wildlife–human interface, wildlife–livestock–human interfaces) is imperative to prevent the emergence of diseases. Adaptation to climate change, and appropriate urban landscape management could help minimize the likelihood of contact between humans, livestock and wildlife [166] (Table 4).

### 5.2. Control Methodologies for Vector-Borne Pathogens

Strategies such as insecticide application, reduction in larval sources, and use of bed nets are of primary importance in order to manage mosquito populations [167]. Biological (for example *Bacillus thuringiensis* and *Wolbachia*) and chemical (insect growth regulators and pyrethroids) approaches have also been used as control methods for invasive mosquitoes [168]. In addition, eliminating mosquito breeding habitats, such as containers and various water cavities, is of utmost importance [169]. It is essential that communities be educated about identifying areas that are conducive to mosquito breeding and the need to take personal protective measures, such as wearing long-sleeved clothing and using insect repellents (Table 5) [146].

## 6. Knowledge Gaps and Future Directions

Despite significant advances in understanding zoonotic spillover and viral emergence, there are still several critical knowledge gaps that hinder effective pandemic prediction and prevention. For example, the lack of a complete understanding of the biological mechanisms underlying the species barrier is one of the fundamental limitations. Indeed, although phylogenetic proximity, receptor compatibility, and viral genetic variability have been implicated, the precise determinants that allow certain viruses to achieve sustainable human-to-human transmission remain unclear [84,170,171,172]. In particular, the role of viral quasi-species diversity, the ability of certain species to invade and evolve in new tissues and hosts, as well as adaptive mutations that enhance this adaptation and receptor binding or immune evasion, require further investigation [173,174,175,176]. Furthermore, the identification and recording of wildlife reservoirs remain incomplete. Especially in biodiversity-rich areas, many potential reservoir species and their associated viral diversity remain unknown [177,178]. Another obstacle to effective pandemic prediction and prevention is the lack of systematic, longitudinal surveillance studies in high-risk human populations, including individuals with frequent occupational or environmental exposure to animals. In fact, current surveillance efforts are often reactive rather than predictive, limiting their ability to detect early signs of emerging epidemics [179].

Another major challenge is the limited integration of data across the human–animal–environment interface [180]. Although the One Health approach is widely endorsed, its implementation remains fragmented, with insufficient coordination between veterinary, environmental and public health sectors. The absence of unified global data-sharing platforms and standardized surveillance frameworks further limits the possibilities for early detection and rapid response [181,182].

Technological advancements, including artificial intelligence and predictive modeling, offer promising opportunities for improving pandemic preparedness [183,184,185]. Integrating genomic, ecological, and epidemiological data could enable the development of early warning systems capable of identifying high-risk spillover events before widespread transmission occurs [181,186]. However, these approaches remain underutilized and require substantial investment and international collaboration.

Another major barrier to effective pandemic prevention is global inequities. Indeed, despite the fact that low- and middle-income countries are usually at the epicenter of zoonotic spillover events, they often lack the infrastructure for genomic surveillance, diagnostic capacity, and vaccine development [187,188,189]. Addressing these inequities is essential, not only for local health systems but also for global health security [190].

Last but not least, the translational gap that persists between scientific knowledge and policy implementation [191]. Although the drivers of zoonotic spillover are increasingly well characterized, this knowledge is not consistently translated into effective, evidence-based interventions. Bridging this gap will require stronger collaboration between scientists, policymakers, and international organizations, as well as sustained political commitment and investment [192].

Moving forward, shifting from a reactive to a predictive and prevention-oriented framework is essential. This will require a multidisciplinary, globally coordinated effort that combines mechanistic research, technological innovation, and equitable public health strategies to mitigate the risk of future pandemics [193].

### Structured Operational Actions

To achieve true prevention of the next global health crisis, the international scientific and policy communities must proceed with structured operational actions:

1. Map Molecular Mechanisms of the Species Barrier

- Identify Receptor Co-evolution: Fund deep-sequencing and structural biology projects focusing on how viral surface glycoproteins bind to animal vs. human cellular receptors [194].
- Profile Tissue Tropism: Map downstream intracellular restrictions (such as host polymerase inhibitors or innate immune system evasion pathways) that limit replication in human cells.

2. Standardize Hotspot and Reservoir Catalogs

- Target Biodiversity Hotspots: Direct molecular screening teams explicitly to primary tropical forest borders, land-use change zones, and active agricultural frontiers [195].
- Build Non-invasive Bio-banks: Systematically archive fecal, saliva, and environmental DNA (eDNA) samples from bats, rodents, and wild non-human primates to uncover hidden viral diversity without harming native ecosystems [196].

3. Deploy Long-term Cohort Surveillance

- Monitor High-Risk Interfaces: Establish ongoing, multi-year medical monitoring of forest workers, miners, livestock handlers, and wildlife trade market vendors [197].
- Utilize Multiplex Serology: Run regular pan-viral serological panels on these cohorts to catch unrecognized, mild, or self-limiting spillover events before human-to-human transmission evolves [198].

4. Implement True One Health Frameworks

- Merge Multi-Sector Data: Create computational platforms that combine ecological tracking data (deforestation rates, wildlife migration patterns, climate anomalies) with veterinary diagnostics and human hospital admission feeds [199].
- Co-locate Sampling Networks: Ensure that when a wildlife disease cluster or unexpected die-off is investigated, local livestock and adjacent human communities are simultaneously sampled.

5. Standardize Global Protocols and Data Architectures

- Enact Open-Access Frameworks: Build globally accessible, secure digital sequence repositories that guarantee immediate, unhindered sharing of genetic data during suspected outbreaks [199].
- Harmonize Metadata Standards: Enforce universal protocols for describing sample locations, host species, environmental metadata, and clinical definitions across different nations.

6. Correct Global Funding and Capacity Inequities

- Incentivize Sovereign Cloud/Lab Infrastructure: Provide direct financial and technical investments to build advanced genomics labs, training facilities, and localized data hubs directly inside resource-limited, high-risk regions.
- Reform Benefit-Sharing Mechanisms: Guarantee that the global South nations providing raw viral surveillance data receive early, equitable, and low-cost access to any resulting diagnostics, vaccines, or therapeutics [200].

7. Bridge the Science-to-Policy Translational Gap

- Formulate Measurable Indicators: Convert abstract ecological risk models into clear, actionable metrics (e.g., specific boundaries on wildlife trading or thresholds for deforestation) that lawmakers can legally enforce.
- Mandate Transdisciplinary Advisory Panels: Embed permanent One Health scientists directly into national security, environmental protection, and financial planning ministries rather than leaving them isolated inside public health agencies [201].

## 7. Conclusions

In conclusion, the trajectory of a zoonotic spillover expanding into an outbreak, epidemic, or pandemic hinges entirely on a pathogen’s ability to cross the species barrier and establish sustained, chain-of-transmission cycles within the human population. Preventing these catastrophic global health crises requires an operational understanding of the intersection between pathogen traits, host vulnerabilities, and specific environmental transmission routes.

To effectively mitigate these risks, national governments and global health organizations must transition from reactive containment to proactive prevention by implementing the following concrete actions:

- Targeted Surveillance: Deploy continuous genetic sequencing at high-risk animal–human interfaces, such as live animal markets and deforestation frontiers, to detect early pathogen mutations.
- Integrated Data Sharing: Establish unified, real-time global databases that link veterinary, environmental, and human health data to identify spillover flags before widespread transmission occurs.
- Infrastructure Investment: Strengthen local healthcare infrastructure in spillover hot spots to ensure rapid diagnostics, immediate isolation capabilities, and swift decentralized containment responses.
- One Health Frameworks: Institutionalize collaborative policies across environmental, agricultural, and public health sectors to regulate wildlife trade and preserve natural habitats, directly reducing human–animal exposure.

## Figures and Tables

**Table 1:** Main viral zoonoses.

Virus	Reservoir	Intermediate Host	Vector	Transmission to Humans	Ref
Marburg	Fruit bats	Non-human primates *(amplifying)*	None	Contact with wildlife/fluids	[36]
Ebola	Fruit bats	Non-human primates *(amplifying)*	None	Contact with wildlife/fluids	[37]
SIV	Old world primates	None	None	Wildlife handling and consumption	[38]
Hepatitis E	Domestic pigs and wild boars	None	None	Fecal-oral route, pork consumption	[39]
Swine influenza viruses (Type A, subtypes H1N1, H1N2, H3N2)	Pigs	None	None	Direct contact (pig farmers and pork producers)	[40]
Rota viruses G3, G5 and G9 (Group A Rotavirus)	Pigs	None	None	Fecal-oral, contaminated soil and water	[41]
Hendra	Fruit bats	Horses *(amplifying)*	None	Contact with infected horse fluids	[42]
Nipah	Fruit bats	Pigs *(amplifying)*	None	Contact with infected pig/bat fluids or contaminated food	[43]
Hantaviruses	Rodents	None identified	None	Direct contact or inhalation of aerosolized rodent secretions	[44]
Lassa	Rodents (*Mastomys*)	None identified	None	Direct contact or inhalation of aerosolized rodent secretions	[45]
Mpox	Small mammals (e.g., squirrels, rodents)	None required	None	Contact with infected animal lesions, body fluids or carcasses.	[46]
Rift Valley fever	Mosquitoes	Domestic ruminants	Primarily *Aedes* mosquitoes	Vector-borne and handling livestock tissue	[47]
West Nile	Birds (primarily passerine/songbirds)	None (mammals *dead-end*)	*Culex* mosquitoes	Vector-borne	[48]
Zika	Humans/Primates	None	*Aedes* mosquitoes (*Aedes aegypti* and *Aedes albopictus*)	Vector-borne	[49]
Yellow fever	All primates	None	Primarily *Aedes* mosquitoes	Vector-borne	[50]
Dengue	Humans/Primates	None	*Aedes* mosquitoes (*Aedes aegypti* and *Aedes albopictus*)	Vector-borne	[51]
Chikungunya	Humans, other primates, rodents, birds	None	*Aedes* mosquitoes (*Aedes aegypti* and *Aedes albopictus*)	Vector-borne	[52]
Ross River	Marsupials	None	Over 40 species of mosquitoes (mainly *Aedes* and *Culex* species)	Vector-borne	[53]
Japanese encephalitis	Wading birds (herons, egrets)	Pigs are the most important amplifying hosts	*Culex* mosquitoes	Vector-borne	[54]
MERS-CoV	Bats	Dromedary camels	None	Direct contact or consuming of raw/unpasteurized camel products	[55]
SARS-CoV-1	Bats	Masked palm civets, raccoon dogs	None	Direct contact with infected animals or their secretions	[56]
SARS-CoV-2	Bats (likely)	Unverified (pangolins and civets remain unconfirmed hypotheses)	None	Direct contact with infected animals or their secretions	[57]
Influenza A, subtype H5N1	Wild waterfowl (ducks, geese, shorebirds)	Domestic poultry/Dairy cattle (act as highly susceptible spillover hosts)	None	Direct contact with sick/dead birds, infected livestock or contaminated environments (feces, litter)	[58]

SIV: simian immunodeficiency virus, MERS-CoV: Middle East respiratory syndrome coronavirus, SARS-CoV: Severe acute respiratory syndrome coronavirus.

**Table 2:** Pandemics from past centuries to the present.

Pandemic	Virus	Years	Estimated Deaths	Ref.
“Influenza” pandemic	Unknown (The influenza virus was identified in 1930s)	1510	Unknown (Death registration began in 1837)	[70]
“Influenza” pandemic	Unknown (The influenza virus was identified in 1930s)	1557–1559	Unknown (Death registration began in 1837)	[71]
“Influenza” pandemic	Unknown (The influenza virus was identified in 1930s)	1580	Unknown (Death registration began in 1837)	[71]
“Influenza” pandemic	Unknown (The influenza virus was identified in 1930s)	1729–1733	Unknown (Death registration began in 1837)	[71]
“Influenza” pandemic	Unknown (The influenza virus was identified in 1930s)	1761	Unknown (Death registration began in 1837)	[71]
“Influenza” pandemic	Unknown (The influenza virus was identified in 1930s)	1775	Unknown (Death registration began in 1837)	[72]
“Influenza” pandemic	Unknown (The influenza virus was identified in 1930s)	1781–1782	Unknown (Death registration began in 1837)	[71]
“Influenza” pandemic	Unknown (The influenza virus was identified in 1930s)	1830–1833	Unknown (Death registration began in 1837)	[73]
“Influenza” pandemic	Unknown (The influenza virus was identified in 1930s)	1847–1851	Unknown	[74]
“Russian flu”	Unknown (The influenza virus was identified in 1930s)	1889–1890	~1 million	[75]
“Spanish flu”	Influenza A virus, subtype H1N1	1918–1919	>50 million	[76]
“Asian flu”	Influenza A virus, subtype H2N2	1957–1958	~1–2 million	[76]
“Hong Kong flu”	Influenza A virus, subtype H3N2	1968–1969	~¾ million	[77]
“Russian flu”	Influenza A virus, subtype H1N1	1977–1979	~700.000	[78]
HIV/AIDS pandemic	HIV	1981–present	~45 million (until 2026)	[79]
“Swine flu”	Influenza A virus, subtype H1N1	2009–2010	Laboratory-confirmed deaths: 18.449 officially reported to the WHO by mid-2010 *	[77]
COVID-19 pandemic	COVID-19	2019–present	~7.11 million confirmed deaths (until 2026) **	[80]

HIV: Human immunodeficiency virus, AIDS: Acquired immunodeficiency syndrome, COVID 19: Coronavirus disease 2019. * The US Centers for Disease Control and Prevention (CDC) and the WHO estimated that the true number of deaths was actually between 151,700 and 575,400 people worldwide in the first year alone. This huge difference is due to the fact that laboratory tests were not widely available or performed on everyone during the pandemic. Many deaths associated with the H1N1 virus occurred in areas with limited health care resources (particularly in Southeast Asia and Africa) or were classified as cardiovascular or respiratory complications rather than direct H1N1 infections [81]. ** The actual number of deaths may be much higher than the number of confirmed deaths shown here, due to limited data from some countries on testing and medical records as well as death registries.

**Table 3:** Decoding Zoonotic Spillover.

Phase	Pathogen Factors	Host Factors	Human Activities
**1. Initial Cross-Species Jump**	Ability to bind human receptors Ability to invade human cells Overcoming species barriers	Frequent animal contact High-risk occupation Poor host immune status Genetic predisposition Lack of vaccination Extreme age (young/old) Underlying health status	Wildlife hunting and handling Wildlife butchering and carcass disposal Ecotourism and wildlife safaris Direct contact with bats and rodents Contact with infected bird feces/litter Inhaling aerosolized rodent secretions Commercial bushmeat trade Wet markets and exotic food handling Keeping exotic pets
**2. Within-Host Adaptation**	Antigenic drift and shift Genetic reassortment capacity Immune escape mechanisms		
**3. Human-to-Human Spread**	Easy human-to-human transmission Low host mortality rates Long-term chronic infections	Low population immunity High-density living conditions	Poor personal hygiene practices Intensive animal husbandry practices

**Table 4:** Wildlife spillover interfaces: interventions and major challenges.

Interface	Key Stakeholders/Target Areas	Key Interventions	Major Challenges
**Occupational**	Hunters, Foresters, Zoo Workers	Targeted safety training. Mandatory vaccinations (e.g., rabies). Provision of personal protective equipment (PPE) (gloves and masks).	Poverty, lack of clean water, and deeply ingrained cultural livelihood dependencies frequently limit compliance.
**Local Market**	Traders, Consumers, Domestic Animals	Strict market hygiene rules. Physical separation of diverse species. Consumer education on thorough cooking processes.	Standard hygiene guidelines fail without strict enforcement. Economic pressures in low-income regions bypass rules. Species separation is difficult to sustain without top-down market restructure.
**Global Trade**	Importers, Exporters, Border Control	Airport and border screening. Real-time data sharing and regulation alignment. Intergovernmental surveillance body funded by high-income countries.	High-income importing nations drive macroeconomic dependencies. Severe lack of global equity in funding and resource distribution. Import bans risk pushing the wildlife trade underground rather than mitigating it.
**Ecological and Urban-Agricultural**	Urban Planners, Farmers, Displaced Wildlife	Urban buffers and infrastructure design. Strict land-use zoning against forest encroachment. Livestock biosecurity barriers.	Deforestation and fragmented landscapes create dangerous “edge effects”. Interventions require massive, cross-sectoral One Health cooperation. Commercial pressure from agriculture, logging, and development limits success.

**Table 5:** Strategic mosquito control interventions across spillover interfaces.

Interface	Focus and Target	Interventions
**Environmental and Sylvatic Interface** (Source Control)	Preventing mosquito amplification in natural and semi-natural habitats before they interact with human populations	**Biological control:** *Bacillus thuringiensis*, *Wolbachia.* **Environmental management:** Larval source reduction (eliminating stagnant water) and habitat elimination (removing containers, puddles, and water cavities around residential areas).
**Peri-Domestic and Agricultural Interface** (Buffer Zones)	Targeting transition zones where invasive vectors breed in man-made environments and interact with livestock, working humans, and residential borders.	**Larval source reduction** and container management. **Insect growth regulators** (IGRs).
**Domestic and Urban Interface** (Direct Human Transmission)	Securing the immediate living space where the probability of vector-to-human contact and pathogen spillover is highest.	**Vector control:** Chemical control (Insecticide application, Pyrathroids, IGRs) and biological control (*Bacillus thuringiensis*, *Wolbachia*). **Personal and community measures:** Personal protective measures (Bed nets, repellents, long-sleeved clothing) and community education.

## Data Availability

No new data were created or analyzed in this study. Data sharing is not applicable.
